# Targeting c-MYC: a potential non-hormonal therapeutic approach for endometriosis treatment

**DOI:** 10.3389/fcell.2023.1225055

**Published:** 2023-11-21

**Authors:** Warren B. Nothnick, Sachith Polpitiya Arachchige, Paige Minchella, Edward B. Stephens, Amanda Graham

**Affiliations:** ^1^ Department of Cell Biology and Physiology, University of Kansas Medical Center, Kansas City, KS, United States; ^2^ Department of Obstetrics and Gynecology, University of Kansas Medical Center, Kansas City, KS, United States; ^3^ Department of Cancer Biology, University of Kansas Medical Center, Kansas City, KS, United States; ^4^ Center for Reproductive Sciences, Institute for Reproductive and Developmental Sciences, University of Kansas Medical Center, Kansas City, KS, United States; ^5^ Department of Microbiology, Molecular Genetics and Immunology, University of Kansas Medical Center, Kansas City, KS, United States

**Keywords:** c-Myc, endometrial cancer, endometriosis, Omomyc, treatment

## Abstract

Endometriosis is a benign gynecological disease in which eutopic endometrial tissue composed of glands and stroma grow within the pelvic cavity. The disease affects females of reproductive age and is characterized by pelvic pain, infertility and reduced quality of life. The majority of pharmacologic treatment modalities for endometriosis focus on suppression of estradiol production and/or action; an approach associated with adverse side effects. c-MYC is elevated in eutopic endometrium and endometriotic lesion tissue in patients with endometriosis and the disease shares many similar pathological characteristics with that of endometrial carcinoma. While targeting of c-MYC with Omomyc has recently gained substantial interest in the field of cancer research, there has been no recent attempt to evaluate the potential utility in targeting c-MYC for endometriosis treatment. The following perspective article compares the similarities between endometriosis and endometrial cancer and presents preliminary data suggesting that targeting c-MYC with Omomyc reduces endometriotic cell proliferation and viability *in vitro*. Future application of targeting c-MYC in endometriosis treatment and potential pros and cons are then discussed.

## 1 Introduction

Endometriosis is a chronic inflammatory disease in which endometrial tissue grows outside the uterine cavity, predominantly within the pelvic cavity (Giudice and Kao, 2004). The disease affects approximately 10%–15% of women of reproductive age and is associated with pelvic pain, dysmenorrhea, infertility, and reduced quality of life (Giudice and Kao, 2004). The etiology of endometriosis is not clear although retrograde menstruation is widely accepted (Sampson, 1927). It is postulated that menstrual overflow leaves the uterine cavity through the fallopian tubes and implants into the peritoneal cavity and the ovaries. However, most women experience retrograde menstruation, yet not all women develop the disease (Jenkins et al., 1986). Therefore, multiple factors contribute to the development and progression of endometriosis such as aberrant immune response and altered hormonal balance (Sourial et al., 2014; MacLean and Hayashi, 2022). Given that the growth of endometrial lesions is estrogen-dependent, common pharmacologic treatments target estrogen production and subsequent estrogen action and include oral contraceptive pills (OCPs), gonadotropin-releasing hormone (GnRH) agonists, GnRH antagonists, levonorgestrel (progestin)-releasing intra-uterine devices and aromatase inhibitors (Nothnick et al., 2018). We discuss below the current pharmacologic approaches for endometriosis treatment and their limitations. We then discuss the similarities between endometriosis and endometrial cancer and review targeting of Myc as a therapeutic approach in cancer treatment. Lastly, we provide preliminary evidence supporting the potential of targeting c-MYC as a non-hormonal treatment option for endometriosis.

## 2 Current approaches to endometriosis treatment

Oral contraceptive pills (OCPs), which contain low dose estrogens and high dose progesterone, are often the first line approach in treating endometriosis/endometriosis-associated pain (Menakaya et al., 2013; Dunselman et al., 2014). Low levels of estrogen are postulated to induced progesterone receptor expression and the progesterone/progestins contained within the OCP preparations inhibit estrogen production by the ovaries via suppression of gonadotropin release as well as via reduction of the inflammatory milieu associated with the disease. Progestins such as medroxyprogesterone acetate (MPA) are also effective in controlling pain, but a drawback associated with their use is side effects such as menstrual irregularities and weight gain (Brown et al., 2012). Levonorgestrel-releasing intrauterine system (LNG-IUS) is a popular treatment option effective in reducing dysmenorrhea, pelvic pain, deep dyspareunia as well as reducing lesion burden (Fedele et al., 2001; Bayoglu et al., 2011; Kim et al., 2022) and can be used as a long-term treatment option (Kim et al., 2022). While LNG-IUS offers many benefits, limitations include irregular uterine bleeding, vaginal bleeding and vaginal discharge. In summary, LNG-IUS treatment is an effective and feasible method to control pain as a long-term postoperative maintenance therapy for endometriosis patients. While progestin-based therapies are effective in many women, a substantial percentage of endometriosis patients exhibit progesterone resistance and therefore have insufficient therapeutic responses to these treatments.

The fact that endometriosis is an estrogen-dependent disease led to targeting production of this hormone as a means of treating the disease. Targeting gonadotropin release at the level of the hypothalamus to reduce circulating estrogen levels has proven effective and overcomes the issue of progesterone resistance. Gonadotropin hormone releasing analogs includes the use of both gonadotropin-releasing hormone (GnRH) agonists and, more recently antagonists. Both classes of drugs suppress ovarian estrogen production leading to a hypo-estrogenic state which is detrimental to endometriotic lesion survival. GnRH analogs are often prescribed when oral contraceptives and/or progestin analogues fail to produce successful outcomes. These compounds are effective in some, but not all women with endometriosis (Shaw, 1990). Further, GnRH agonist use is associated with significant side effects including altered lipid profile, hot flushes, loss of libido and reduction in bone mass/bone health (Prentice, 2001). The loss of bone mass can be overcome by estrogen add-back/hormone replacement therapy (Surrey, 2010) but this must be balanced to avoid estrogen action upon lesion survival and potential recurrence of disease and its symptomology.

One of the most common and successful therapies for endometriosis pain management is the GnRH agonist, leuprolide acetate (Geisler et al., 2004). However, while this is an advantage of GnRH agonist therapy, a disadvantage is the induction of a hypo-estrogenic state and negative impact on overall patient health. GnRH antagonists have also been used for treatment of endometriosis. Unlike GnRH agonists, they do not exhibit agonistic effects inducing estrogen “flare-up” prior to suppressing estrogen levels. Elagolix, relugolix, and linzagolix are three recent GnRH antagonist being used for endometriosis treatment (Rzewuska et al., 2023). Unlike earlier formulations, these three antagonists are taken orally, with both elagolix and relugolix already approved by the FDA and linzagolix currently under review. These GnRH antagonists may offer several benefits over older drug formulations which include lower levels of analgesics taken, significant reduction in pain scores and little to no irregular uterine bleeding. However, these GnRH antagonists are still associated with side effects including reduction in bone mineral density and risk of developing osteopenia and osteoporosis due to their induction of a hypoestrogenic state which limits their treatment regime duration (Rzewuska et al., 2023). It should be noted that combination therapy of relugolix with estradiol and norethisterone acetate for 24 weeks minimizes bone density loss and vasomotor symptoms while significantly reducing endometriosis associated pelvic pain (Giudice et al., 2022).

The use of aromatase inhibitors in the treatment of endometriosis have gained attention based upon the observation that endometriotic lesions express aromatase and are able to synthesize their own estrogen (Bulun et al., 2000). Current aromatase inhibitors prescribed include anastrozole and letrozole. Letrozole in combination with the synthetic progesterone analog, norethindrone acetate, was first reported to reduce disease burden at second look laparoscopy as well as significantly reduce pelvic pain scores in 2004 by Ailawadi and colleagues. Subsequent studies continue support the efficacy of letrozole in treatment of endometriosis-associated pelvic pain. Like letrozole, anastrozole, also inhibits estrogen production but the former is more potent in reducing levels of this steroid (Geisler et al., 2002). Anastrozole therapy combined with oral contraceptives was reported to significantly reduce pain in as little as 1 month after treatment initiation and this treatment regime was associated with minimal side effects (Amsterdam et al., 2005). However, the safety of aromatase inhibitors might be an issue especially since a recent systematic review and meta-analysis suggested an increased risk of cardiovascular events during endocrine therapy for early breast cancer (Yoo et al., 2023). None the less, aromatase inhibitors offer an additional treatment modality which is effective in treating endometriosis-associated pelvic pain.

In summary, the majority of currently prescribed endometriosis treatments rely upon reduction of estrogen production and/or estrogen action. As emphasized earlier in this article, while these treatments are effective in many women, they are not effective or well-tolerated in a large proportion of women suffering from endometriosis. These observations coupled with limitations due to side-effects and potential health complications emphasize the need for the development of novel, estrogen-sparing endometriosis treatments. In searching for such novel treatment targets, we next evaluate and compare the common mediators and mechanisms between endometriosis and cancer with the goal of identifying potential druggable targets which may be capable of reducing disease burden and symptomology associated with endometriosis.

## 3 Endometriosis and cancer

**FIGURE 1:**
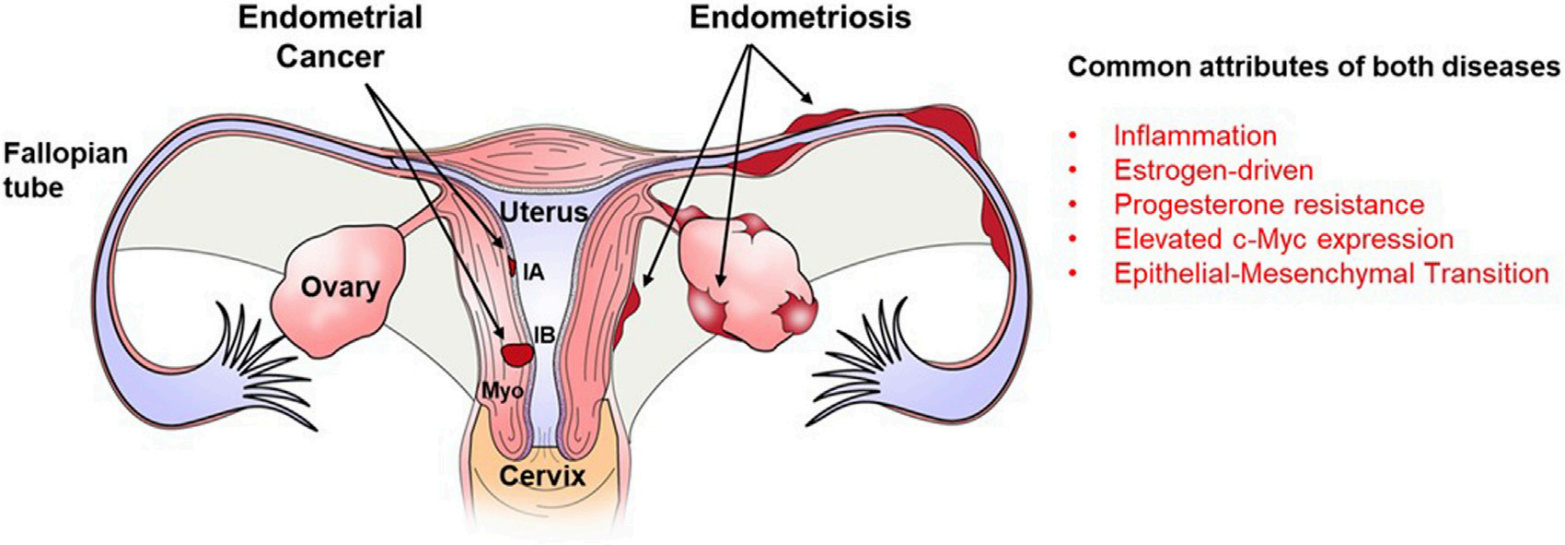
Localization of endometriotic implants and endometrial carcinoma within the female reproductive tract and major similarities between both diseases. Both endometrical cancer and endometriosis originate from the endometrial lining of the uterus. Endometrical cancer which remains confined to the endometrial lining is classified as stage IA while that which spreads into the myometrial smooth muscle (Myo) of the uterus is classified as stage IB. Endometriotic lesions develop outside of the uterus on the surface of the Fallopian tube, ovary and perimetrium of the uterus. Both diseases are characterized by inflammation, estrogen dominance, progesterone resistance, epithelial-mesenchymal transition and elevated expression of the oncogene. c-Myc.

Both endometriosis and endometrial cancer share numerous risk factors and common pathophysiological characteristics (Figure 1). Endometrial cancer is the most commonly diagnosed form of gynecological cancer in developed nations, accounting for approximately 5% of all cancers diagnosed in women (Contreras et al., 2022). Similar to the origins of endometriotic lesions, endometrial cancer originates in the endometrium and development and progression of both diseases is associated with estrogen exposure (Yu et al., 2015). With respect to endometrial cancer, one mechanism by which estrogen may promote progression of endometrial cancer is through the activation of the NLPR3 inflammasome (Liu et al., 2019), which has also recently been proposed to play a role in the pathophysiology of endometriosis (Irandoost, et al., 2023). Like endometriosis (Ailawadi et al., 2004; Patel et al., 2017), endometrial cancer also exhibits progesterone resistance (Gunderson et al., 2012) and displays altered expression of progesterone receptors (Saito et al., 2006; Jongen, et al., 2009).

It is further postulated that the hyper-estrogen and hypo-progesterone milieus associated with both disease contribute to the enhanced proliferation and invasiveness of the endometriotic lesions/endometrial cancer. Epithelial to mesenchymal transition (EMT) is a process by which epithelial cells lose polarity and cell-to-cell contacts, undergo remodeling of the cytoskeleton, and acquire migratory abilities and a mesenchymal-like gene expression program. The EMT process is proposed to play a role in the pathophysiology of both endometrial cancer (Colas et al., 2012; Mirantes et al., 2013) and endometriosis (Yang and Yang, 2017; Konrad et al., 2020) as both diseases are associated with the migration of endometrial cells into surrounding tissues as the diseases progress. One transcription factor whose overexpression induces EMT (Qiu et al., 2016) as well as immune evasion, angiogenesis, ECM remodeling, cell migration and invasion (Masso-Valles and Soucek, 2020) is c-MYC which is overexpressed in both endometriosis and endometrial cancer.

## 4 c-MYC overexpression in endometriosis and endometrial cancer

The Myc family of transcription factors is composed of c-MYC, N-MYC and L-MYC (Adhikary and Eilers, 2005) whose expression is dysregulated in over 70% of human cancers and associated with poor prognosis (Wang et al., 2021). Like other cancer types, c-MYC is highly expressed in endometrial tumors (Kim et al., 2013) and immunohistochemical localization studies revealed a 78.3% positive rate of c-MYC in endometrial cancer tissues with amplified *c*-*MYC* in 25% of the cases (Zhang et al., 2018; Buchynska et al., 2019). From a functional standpoint, upregulation of c-MYC in endometrial cancer cells *in vitro* was shown to induce EMT, drug resistance and invasion (Lv et al., 2012; Liu et al., 2015). Qiu and colleagues (2016) used a small molecule bromodomain 4 (BRD4) inhibitor, JQ1, to target c-MYC in endometrial cancer cells using both cell culture and tumor tissue xenograft models. In that study, JQ1 inhibited endometrial cancer growth in both models and this was associated with a reduction in c-MYC protein expression as well as reduced expression of c-MYC downstream targets. Additional studies demonstrated that inactivation of c-MYC resulted in tumor regression and was associated with cellular differentiation and apoptosis in transgenic mouse models (Jain et al., 2002; Tansey, 2014). Lastly, a more recent study using JQ1 demonstrated that JQ1-mediated reduction of c-MYC was associated with suppressed tumor growth in a xenograft mouse model as well as reduced proliferation and enhanced apoptosis of endometrial carcinoma cell lines *in vitro* (Pang et al., 2022). Thus, in addition to being a well-established target in multiple types of cancer, c-MYC also appears a plausible target for endometrial carcinoma.

Similar to expression levels in endometrial carcinoma, c-MYC expression is also elevated in endometriotic lesion tissue as well as matched eutopic endometrium from women with endometriosis (Schenken et al., 1991; Schneider et al., 1998; Johnson et al., 2005; Pellegrini et al., 2012; Proestling et al., 2015). Unfortunately, outside of these descriptive studies, little advancement has been made on our understanding as to why c-MYC is elevated in endometriotic lesion tissue and if this overexpression contributes to the pathophysiology of the disease. Based upon the similarities between endometrial cancer and endometriosis and the fact that c-MYC appears to a common transcription factor with augmented expression and signaling in both endometrial cancer and endometriosis, it may also be a viable, non-hormonal treatment for endometriosis. To date, the potential utility of targeting c-MYC as a therapeutic approach for endometriosis treatment has not been reported.

## 5 c-MYC as a therapeutic target for endometriosis treatment

**FIGURE 2:**
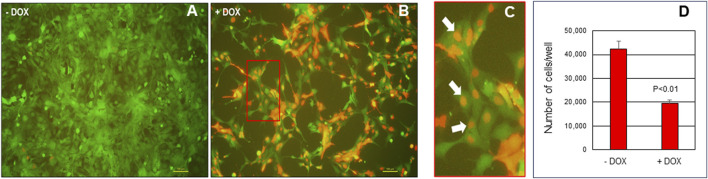
Induction of Omomyc in endometriotic epithelial 12Z cells reduces cell viability. 12Z cells were plated at 25,000 cells/mL of media for 24 h then cultured in the absence of doxycycline ‐ DOX; **(A)** or presence of + DOX; **(B)** doxycycline to induce Omomyc expression (detected by the RFP tag). Red boxed area in panel B is enlarged and displayed in **(C)** with white arrows highlighting nuclear expression of Omomyc. Cell counts were then determined by manual counting and results are depicted in **(D)**. Cell count data were analyzed by unpaired t‐tests on three separate replicates independent experiments (N = 3) and are displayed as the mean +/‐ SEM with P < 0.01.

Given the aforementioned similarities between endometrial cancer and endometriosis and the necessity to identify novel, non-hormonal targets for endometriosis treatment, we conducted the following preliminary studies. To begin to evaluate potential targeting of c-MYC in endometriosis therapy, we utilized the well-characterized human endometriotic epithelial cell line, 12Z which expressed eGFP (Bulun et al., 2000) to evaluate the impact of blocking c-MYC signaling on cell proliferation and survival. To do so, 12Z-eGFP cells were transduced with lentiviral particles expressing a doxycycline (DOX)-inducible pTRIPZ-Omomyc-RFP (Omomyc) plasmid (Annibali et al., 2014) and were then subjected to puromycin-selected. Puromycin resistant Omomyc plasmid-infected 12Z cells (25,000 cells/mL) were cultured in Dulbecco’s Minimum Essential Medium (DMEM)/Ham’s F12 (Fisher Scientific, Pittsburgh, PA) containing 10% TET-FBS and Pen-Strep for 24 h in 10 cm tissue culture dishes after which the media was replaced with fresh media containing 2% TET-FBS with or without doxycycline (DOX, 2.0 μg/mL; Takara Bio catalog# 631311) to induce Omomyc expression. Cells were incubated for 48 h after which they were trypsinized and cell counts determined using a hemocytometer and all counts were conducted on duplicate dishes for 3 separate trials (N = 3). Compared to controls (-DOX), DOX induction (+DOX) of Omomyc resulted in a significant reduction in number of cells after 48 h of treatment. Figure 2 depicts representative immunofluorescence for Omomyc localization with white arrows highlighting nuclear expression of Dox-induced Omomyc expression. Based upon these preliminary studies, Omomyc reduces endometriotic epithelial cell survival *in vitro*. These preliminary observations are encouraging and may warrant further investigation into use of Omomyc to treat endometriosis and its associated symptomology using three-dimensional *in vitro* cell culture models and *in vivo* mouse models of experimental endometriosis routinely employed in our laboratory (Alali et al., 2020).

## 6 Potential c-MYC signaling pathways common to endometrial cancer and endometriosis

To interrogate potential down-stream pathways relevant to c-MYC signaling in the pathogenesis of endometriosis, we generated a list of c-MYC targets which have been reported in cancer (Zeller et al., 2003) and endometrial adenocarcinoma; Peterson et al., 2023) to those reported to be dysregulated in a similar manner in endometriosis. Down-stream targets of c-MYC relevant to endometriosis pathophysiology may include upregulated targets such as cyclin E1 (CCNE1; Park et al., 2019; Park et al., 2020), enolase 1 (ENO1; Nabeta et al., 2009), fatty acid synthase (FASN; Turathum et al., 2022), lactate dehydrogenase A (LDHA; Zheng et al., 2021), microsomal glutathione S-transferase 1 (MGST1; Ferrero et al., 2019), 60S acidic riboprotein 1 (RPLP1; Alali et al., 2020), and tumor protein p53 (TP53; Toki and Nakayama, 2000). c-MYC targets which are downregulated in cancer (Zeller et al., 2003) and endometriosis include cyclin dependent kinase 1A (CDKN1A; Kim et al., 2009) and fibronectin 1 (FN1; Holzer et al., 2020).

## 7 Discussion

c-MYC has long been proposed to play a functional role in the pathophysiology of cancer. However, due to c-MYC’s unique properties with respect to a lack of a defined three-dimensional structure, nuclear localization and absence of a targetable enzymatic pocket, targeting c-MYC for cancer treatment has presented a challenge. Omomyc is a mutant basic helix–loop–helix leucine zipper (bHLHZip) domain which acts as a dominant negative and sequesters c-MYC in complexes preventing active transcription of c-MYC target genes while also allowing transcriptional repression (Masso-Valles and Soucek, 2020). Omomyc has been shown to be a potent inhibitor of tumor growth in multiple cancer types in both *in vitro* and *in vivo* studies (Soucek et al., 2004; Soucek et al., 2008; Sodir, et al., 2011; Soucek et al., 2013; Whitfield, et al., 2014; Fiorentino et al., 2016; Alimova et al., 2019; Sodir et al., 2020). Considering the similarities between cancer and endometriosis (Figure 1) and the limitations with current, anti-estrogen based treatment options for endometriosis, we evaluated the potential utility of Omomyc for endometriosis treatment. To do so, we transduced 12Z cells (a well-characterized endometriotic epithelial cell line) with lentiviral particles containing pTRIPZ-Omomyc-RFP (Omomyc) plasmid and treated them with DOX to induce Omomyc expression. Induction of Omomyc was associated with a reduction in cell viability (as reflected in total cell counts) compared to cells not treated with DOX. Although preliminary, these studies are encouraging and warrant further, more detailed studies. One limitation of current endometriosis treatments is the induction of a hypo-estrogenic state and unwanted side effects associated with it. For Omomyc to be an advancement over current therapies, it will be essential to assess if Omomyc could reduce disease burden and symptomology *in vivo* while concurrently avoiding induction of a hypo-estrogenic state. This would be one critical necessary first step in evaluating this c-MYC inhibitor for endometriosis treatment.

## Data Availability

The raw data supporting the conclusion of this article will be made available by the authors, without undue reservation.
